# Optimizing intra-arterial hypothermia scheme for acute ischemic stroke in an MCAO/R rat model

**DOI:** 10.1038/s41598-023-35824-y

**Published:** 2023-06-13

**Authors:** Yuqi Zeng, Lei Hao, Yue Chen, Shuyi Liu, Yong Fan, Zhenhua Zhao, Yinzhou Wang, Qi Chen, Yongkun Li

**Affiliations:** 1 Department of Neurology, Fujian Medical University Union Hospital, Fuzhou, 350001 China; 2 Department of Neurology, Shengli Clinical Medical College of Fujian Medical University, Fuzhou, 350001 China; 3 Department of Neurology, Second Affiliated Hospital of Xuzhou Medical University, Quanshan District, Xuzhou, 221006 Jiangsu Province China; 4 Central Laboratory, Affiliated Fuzhou First Hospital of Fujian Medical University, Fuzhou, 350001 China; 5 Fujian Key Laboratory of Medical Analysis, Fujian Academy of Medical Sciences, Fuzhou, 350001 China; 6 Fujian Key Laboratory of Innate Immune Biology, Biomedical Research Center of South China, College of Life Science, Fujian Normal University, Fuzhou, 350000 China; 7 Fujian Provincial Key Laboratory of Emergency Medicine, Department of Emergency, Fujian Provincial Hospital, Fuzhou, 350001 China

**Keywords:** Neurological disorders, Stroke, Stroke, Neurology

## Abstract

Hypothermia is a promising neuroprotective treatment. This study aims to explore and optimize the intervention scheme of intra-arterial hypothermia (IAH) in a middle cerebral artery occlusion and reperfusion (MCAO/R) rat model. The MCAO/R model was established with a thread that could be retracted 2 h after occlusion. Cold normal saline was injected into the internal carotid artery (ICA) through a microcatheter in different infusion conditions. Grouping followed an orthogonal design (L_9_[3^4^]) based on three critical factors closely associated with IAH: perfusate temperature (4, 10, 15 °C), infusion flow rate (1/3, 1/2, 2/3 blood flow rate of ICA), and duration (10, 20, 30 min), resulting in 9 subgroups (H_1_, H_2_ to H_9_). A myriad of indexes were monitored, such as vital signs, blood parameters, changes in local ischemic brain tissue temperature (T_b_), ipsilateral jugular venous bulb temperature (T_jvb_), and the core temperature of the anus (T_core_). After 24 h and 72 h of cerebral ischemia, cerebral infarction volume, cerebral water content, and neurological function were assessed to explore the optimal IAH conditions. The results revealed that the three critical factors were independent predictors for cerebral infarction volume, cerebral water content, and neurological function. The optimal perfusion conditions were 4 °C, 2/3 R_ICA_ (0.50 ml/min) for 20 min, and there was a significant correlation between T_b_ and T_jvb_ (R = 0.994,* P* < 0.001). The vital signs, blood routine tests and biochemical indexes showed no significant abnormal changes. These findings revealed that IAH was safe and feasible with the optimized scheme in an MCAO/R rat model.

## Introduction

Worldwide, cerebrovascular accidents, predominantly acute ischemic stroke (AIS), are the second leading cause of death and the third leading cause of disability. The crucial hallmark in AIS treatment is to promptly recanalize the occluded artery to rescue the ischemic penumbra^1,2^. Up to 90% of patients treated with endovascular thrombectomy achieved successful reperfusion^3^. However, retrospective metadata studies indicate that more than half of these patients do not show clinical improvements, and some even show aggravated symptoms, which are generally associated with ischemia/reperfusion injury (IRI) in the involved brain tissue^4,5^. The mechanism driving these outcomes is unclear, but IRI may aggravate blood–brain barrier leakage and brain edema or increase hemorrhagic transformation. Therefore, exploring effective neuroprotective measures targeting IRI for AIS patients is imperative.

Over the last four decades, various animal stroke models have been developed to understand the mechanisms underlying cerebral ischemia and discover new therapeutic strategies^6^. Due to the cerebral vasculature and physiology similarities between rats and humans and ease of operation in animal experiments, rats are generally the first choice in stroke research. The middle cerebral artery occlusion (MCAO) stroke model is the most widely used animal model for human AIS, as it closely resembles the natural causes of disease and consequent neuropathology^4,6,7^. Many animal studies have shown that some neuroprotectants, *e.g.,* recombinant Erythropoietin, nerve growth factor, NXY-059, or neuroglobin, have strong positive neuroprotective activity but never translated into clinical trials^8,9^. Many factors can determine the progression of novel therapeutics to the clinic, but the pathophysiological complexity of IRI involving various cells and molecular mechanisms significantly hinders the discovery of successful therapies. Current neuroprotectants usually target a particular node of this pathophysiological network, struggling to inhibit brain tissue damage driven by multiple redundant signaling cascades. Therapeutic hypothermia (TH) is a promising neuroprotective measure^10–12^ that may inhibit the IRI network by inhibiting multiple signaling cascades of the pathophysiological process, e.g., reducing cell metabolism and generating of free radicals, and inhibiting protein synthesis. Altogether these might alleviate the destruction and permeability of the blood–brain barrier and ischemic neurons^13,14^.

Systemic hypothermia was difficult to clinical transformation due to obvious side effects and taking longer to reach the target temperature^15^. During the past several years, intra-arterial cerebral hypothermia (IAH) has become possible with the wide application of endovascular thrombectomy (EVT) for LVO-AIS. Hypothetically, IAH has a neuroprotective effect and significantly reduces the incidence of side effects and complications related to TH, rendering it a more effective therapy for AIS. Some published studies on theoretical models hypothesized that perfusion of cold normal-saline (NS) through the carotid artery could quickly reduce the temperature of the local brain and produce a protective effect on ischemic brain tissue with minimal influence on the body temperature^16^, which has been recently tested experimentally in pre-clinical^17,18^ and clinical studies^19,20^. Nevertheless, IAH is far from standardized or optimized, and it is likely that some parameters, such as different perfusate temperatures, flow rate, and duration, would affect local brain tissue and whole body temperatures, leading to significant differences in efficacy and safety. To date, few preclinical or clinical studies have elaborately focused on the executing details of IAH.

Therefore, we will explore and optimize the scheme of IAH in MCAO/R rat models and investigate the temporal changes of local brain tissue temperature (T_b_), jugular venous bulb temperature (T_jvb_), and whole-body core temperature (T_core_) during cold saline perfusion.

## Materials and methods

### Study design

All animal experiments were reviewed, authorized, and conducted following the regulations of the Animal Ethics Committee of Fujian Normal University (Approval No. *IACUC-20210031*). Age-matched, specific pathogen-free, male Sprague Dawley rats (8 weeks old, 240–265 g) were raised according to the experimental animal feeding standards and provided by the Experimental Animal Center of Fujian Normal University.

A three-factor and three-level (L9[3^4^]) orthogonal test design method was adopted in this study to investigate the optimal perfusion conditions with cold NS: perfusate temperature (Factor A) at 4, 10, 15 °C; perfusion flow rate (Factor B) of 1/3, 1/2, or 2/3 R_ICA_ (blood flow Rate of the internal carotid artery (ICA), estimated as 0.75–2.0 mL/min according to the section area^21^ and flow velocity of ICA (V_ICA_)^22^; and perfusion duration (Factor C) of 10, 20, or 30 min. Even though the orthogonal design table does not distinguish the specific interaction between various factors, this approach was selected to identify the best combination of all factors and levels (or the optimal perfusion conditions). The orthogonal design table (Table 1) was equally distributed and divided into 9 therapeutic subgroups (H_1–9_, n = 14). The optimal IAH condition was statistically estimated according to three outcome-related indexes: cerebral infarct volume 24 h after the intervention, cerebral water content, and modified neurological score scale (mNSS) 72 h after intervention.

**Table 1:** The three factors and three levels orthogonal table (L_9_(3^4^)) for IAH in MCAO/R rats.

Subgroup (randomized)	Factor A: Perfusate temperature (℃)	Factor B: Infusion flow rate (R_ICA_*, ml/min)	Factor C: Infusion duration (min)	Total infusion volume (ml)
H_1_	(1) 4	(1) 1/3, 0.25	(3) 30	7.5
H_2_	(1) 4	(2) 1/2, 0.38	(2) 20	7.5
H_3_	(1) 4	(3) 2/3, 0.50	(1) 10	5.0
H_4_	(2) 10	(3) 2/3, 0.50	(2) 20	10.0
H_5_	(2) 10	(2) 1/2, 0.38	(3) 30	11.3
H_6_	(2) 10	(1) 1/3, 0.25	(1) 10	2.5
H_7_	(3) 15	(1) 1/3, 0.25	(2) 20	5.0
H_8_	(3) 15	(2) 1/2, 0.38	(1) 10	11.3
H_9_	(3) 15	(3) 2/3, 0.50	(3) 30	15.0

*IAH* intra-arterial cerebral hypothermia, *MCAO/R* middle cerebral artery Occlusion and Reperfusion, *N*_*i*_ the number of subjects in each subgroup(I = 1–9); H_1–9_ represent 9 subgroups of orthogonal design with different combinations of the 3 factors and 3 levels: Level 1 represents 4 °C, 1/3 R_ICA_, and 10 min; Level 2 represents 10 °C, 1/2 R_ICA_, and 20 min; and Level 3 represents 15 °C, 2 /3 R_ICA_, and 30 min in the factors of perfusate temperature, infusion flow rate, and infusion duration. *R_ICA_, blood flow rate of internal carotid artery (ICA) per minute, which was estimated as 0.75 ml/min referred previous reports^21^.

### Establishing MCAO/R models

**Figure 1:**
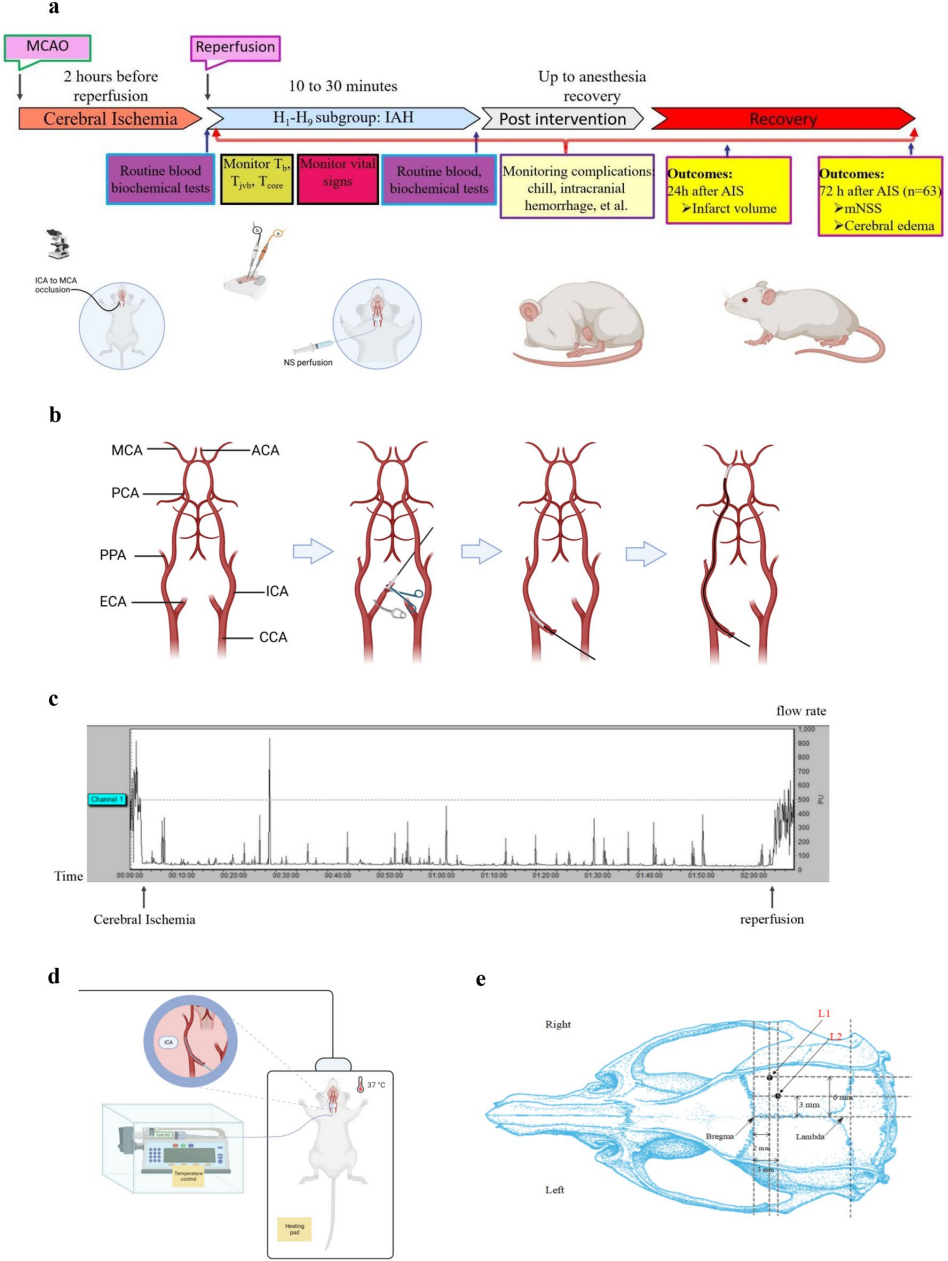
General experimental outlines and procedures in this study. (**a**) Schematics of time points for a surgical procedure, sample collection, and measurement endpoints *(Created with BioRender.com*). (**b**) Anatomical schematic of surgical procedures for MCAO (*Created with BioRender.com*). i: Anatomy of rat's brain arteries; ii: The ECA was ligated and cut open, and a silicone-tipped monofilament was then inserted towards the ECA; iii: The ECA was inverted parallel to the ICA, and monofilament inserted into the ICA; iv: The monofilament was carefully inserted from the ICA to the beginning of the MCA and fixed. (**c**) Laser Doppler detection of the brain blood flow changes of MCAO/R models. The homodynamic criteria for successful modeling were a decrease in the blood supply area of the middle cerebral artery to less than 20% of the preoperative cortical blood flow rate after the insertion of the threaded plug; If the cortical blood flow rate was restored to 80% or more of the preoperative blood flow rate when the threaded plug was removed 2 h after operative, which indicated that the reperfusion was successful. (**d**) Schematics of the protocol of IAH (*Created with BioRender.com*). A 1.5 F microcatheter, guided with a 0.010-inch Micro-guidewire, was inserted into the ICA about 0.8–1.2 cm through the ECA stump. The Micro-guidewire was withdrawn, and the other side of the microcatheter was connected to the syringe pump, which continuously pumped cold NS at a preset rate, temperature. **e** Anatomical Schematic of the incision location for the laser Doppler probe and measuring T_b_. L_1_: The incision site for the laser Doppler probe (bregma/lateral: −2/6 mm (*Paxinos and Watson, 2005*)); L_2_: The incision site for measuring Tb (bregma/lateral/depth: −3/3/−2 mm (*Paxinos and Watson, 2005*)). Notes: CCA, common carotid artery; IAH, Intra-arterial therapeutic hypothermia; MCAO/R, middle cerebral artery occlusion/reperfusion. ICA: internal carotid artery; ECA: external carotid artery; ACA: anterior cerebral artery; MCA: middle cerebral artery; PCA: posterior cerebral artery; PPA: Pterygopalatine artery; OA: occipital artery.

The MCAO/R model in SD rats was established as previously described^22^ with slight modifications (Fig. 1a, b). Rats were fasted for 12 h with access to water ad libitum before operation. Anesthesia was induced and maintained with 1.5–3.5% enflurane in 70% nitrous oxide and 30% oxygen. The right common carotid artery, external carotid artery (ECA), ICA, and the right internal jugular bulb were carefully anatomically uncovered. Under the stereomicroscope (German Carl Zeiss, SteREO Discovery.V8), a 0.4 mm silicone-tipped monofilament (Rayward, Shenzhen, China) was inserted up to 18–20 mm into the internal carotid artery (ICA) through the right external carotid artery (ECA) to occlude the middle cerebral artery (MCA) as shown in Fig. 1b. After 2 h, the silicone-tipped monofilament was retracted to allow reperfusion. Successful occlusion and reperfusion of the MCA were confirmed by a laser Doppler flowmetry probe (British Gene & I, MoorVMS-LDF2) placed on the cranial skull next to the main stem of MCA, which was modified from previously described^23^. Briefly, A small incision (diameter: 1–2 mm) was opened with a thin skull drill needle to expose the right cerebral cortex and place the laser Doppler probe into the incision (bregma/lateral: −2/6 mm) to monitor cortical blood flow, then fixed the probe on the skull by glue (Fig. 1c). Successful occlusion decreased the MCA blood supply area to less than 20% of the preoperative cortical blood flow.

Successful reperfusion was restored to 80% or more of the preoperative blood flow after filament removal and operative practice by Laser Doppler (Gene&I, MoorVMS-LDF2) (Fig. 1c). Model rats were randomized to nine subgroups for IAH. At the end of the surgical procedure, the ECA stump was sutured with a pen-type coagulator, then sewed layer by layer, and 0.25% Bupivacaine (4 mg/kg) was administered to relieve pain. Animals were returned to their cages, with access to food and water ad libitum*,* and tested for cerebral infarction volume in 24 h, cerebral water content, and neurological function(mNSS) in 72 h post-surgery. Subjects with severe intracranial hemorrhage or died after intervention were excluded.

### Perfusion of cold normal saline (NS)

The protocol of IAH was showed in Fig. 1d. Sodium chloride (0.9% v/v) injection was heparinized by heparin sodium injection (5–10 mg/kg) and cooled down to the target temperature before operation. Two hours after occlusion, the filament was carefully removed, and a 1.5 F microcatheter, guided with a 0.010-inch Micro-guidewire, was inserted into the ICA about 0.8–1.2 cm through the ECA stump. The Micro-guidewire was withdrawn, and the other side of the microcatheter was connected to the syringe pump, which continuously pumped cold NS at a uniform rate, temperature (low-temperature thermostat bath kept the liquid temperature at a preset level, i.e., 4, 10, 15 °C) and continuous time. Every rat model within a subgroup will receive the same total perfusate volume calculated by the preset infusion rate and continuous time ($0.{7}5 \times {\text{Rate}} \times {\text{Time}}$) (Table 1). While injecting, the microcatheter was immersed in a thermostat bath with the same preset temperature to maintain a stable perfusate temperature. During the intervention, a thermostatic pad was placed under the rats to maintain body temperature. After perfusion, the microcatheter was withdrawn, the stump of ECA was ligated, and the neck incision was sutured layer by layer.

### Monitoring core and local temperature during perfusion

The temperature was monitored with an ultra-fast precision Thermometer (DAE-905 T, Guangzhou Shenggao, China) and an anal temperature detector. Local T_b_ was measured near the main stem of MCA on the ischemic side at a depth of about 2 mm from the surface of the skull (bregma/lateral/depth: −3/3/2 mm (*Paxinos and Watson, 2005*)), which was regarded as local brain temperature (Fig. 1e). T_jvb_ was detected with the same needle-type Thermometer by penetrating the left JVB. T_b_, T_jvb,_ and T_core_ were measured every three min during and after perfusion until rewarming. Some T_b_-related indexes were observed and analyzed, such as the Time of T_b_ initially reaching below 35 °C (Time of T_b-35 °C_, represents the velocity of T_b_ lowering), the minimum of T_b_ (T_b-min_), and Time of T_b_ being maintained below 35 °C (Time of TH, represents the valid time of therapeutic hypothermia) and Time of rewarming after infusion. The measurement accuracy was 0.01 °C for T_b _and T_jvb_ and 0.1 °C for T_core_.

### Monitoring physiological and biochemical indicators

Animals' vital signs, including anal temperature, heart rate, breathing rate, and blood oxygen saturation (SaO_2_), were monitored with a small animal physiological monitor (STARR, Mouse Ox). Subjects collected 1 ml blood from the left JVB before and after cold saline perfusion for routine blood test (n = 7) with BS-220 automatic multi-species five-category animal blood analyzer (Mindray, China) and tests of biochemical and other indicators with Celercare® M automatic biochemical analyzer (MNCHIP). Surgery-related complications, hypothermia-related side effects and/or arrhythmia, shivering after resuscitation, and intracranial hemorrhage, were observed. Rats would be excluded if they died from surgery or were found with intracranial hemorrhage.

### Measurement of cerebral infarction volume

24 h after cerebral ischemia, the subjects were anesthetic and sacrificed to measure cerebral infarction volume (H_1–9_, n = 7) by the staining method of 2, 3, 5-Triphenyl tetrazolium chloride (TTC)^24^. Brain tissue was frozen at −80 °C for 8–10 min, and 6 coronal brain slices (≈2 mm thick) were cut continuously and equally distanced. Brain sections were stained in freshly prepared 0.1% v/v solution of TTC (Solaibo) and incubated for 20 min in an electrothermal incubator (Shanghai MOMA, DHP-9052) at 37 °C. Samples were turned over once until the normal non-infarcted tissue was stained crimson, and the infarcted tissue appeared white. Histological brain sections were then transferred to 4% paraformaldehyde in phosphate-buffered saline (PBS, Sinopharm Chemical Reagent Co., Ltd.) for fixation and imaged with a Canon camera (ESO 70D). Images were analyzed with Image J (National Institutes of Health) software. A simple integration method was used to measure the volume of cerebral infarction, calculated as V(mm^3^) = A(mm^2^) × d(mm), where "A" was the sum of the infarct area on each brain section, and "d" was the section thickness. The volume of cerebral infarction was normalized to each animal's total brain tissue volume prior to statistical analysis and comparison between subgroups.

### Neurological score

The neurological function was assessed for model rats in every subgroup (H_1–9_, n = 7) in 72 h after cerebral ischemia by the modified Neurological Severity Score (mNSS) as previously described^25^.

### Cerebral water content

Cerebral water content was evaluated after mNSS (H_1–9_, n = 7). Subjects were anesthetic and sacrificed 72 h after cerebral ischemia, and brains were collected as described above, with the olfactory bulb, cerebellum, and pia mater removed. Cerebral water content was detected as in previous reports. Briefly,unsanctioned brains were washed with NS, blotted on filter paper, and weighed for wet brain mass with a precision electronic balance (HY-FA301, Anhui Huabiao Testing Instrument Co. Ltd, China). Unsanctioned brain samples were then dehydrated at 100 °C constant temperature for 24 h on a laboratory oven (DHP-9052, Shanghai MoMa Thermostatic Equipment, China), and brain stem mass was weighed again with a precision electronic balance. Brain tissue's water content was calculated as (wet mass—dry mass)/Wet mass × 100%. Brain tissue's water content was used to measure brain tissue edema.

### Statistical analysis

All statistical analyses were performed using SPSS 21.0 software (IBM Corp., Armonk, NY, USA). Continuous variables are presented as mean ± SD or median (interquartile range, IQR) and were analyzed using Student's t-tests or Mann–Whitney U tests as appropriate. Categorical data were presented as numbers and percentages, which were analyzed using the X^2^ or Fisher's exact tests. A 2-tailed probability value of *P* < 0.05 was considered statistically significant. Among the nine subgroups, the survival rate, the T_b_-related indexes, and the outcome-related variables (cerebral infarction volume, cerebral water content, and mNSS) were analyzed between the best subgroup and the other 8 subgroups by Dunnett's *t*-test. The better factor and level were primarily identified by comparing the range of level means within the same factor. Then, the rank of factors and their corresponding best level were further verified by the orthogonal test's analysis of Variance (ANOVA) and the least significant difference (LSD) method. The correlation between T_b_ and T_jvb_ was analyzed by Pearson correlation analysis.

### Ethics approval

All experimental procedures were approved by the Animal Ethics Committee of Fujian Normal University (Approval No. *IACUC-20210031*), and efforts were made to reduce the total number of animals used and their potential pain and suffering. This study was reported following ARRIVE guidelines.

## Results

### Physiological and biochemical indexes and complications during IAH

One hundred and fifty-two rats were finally used, 26 were excluded due to dying from surgery (14.47%, 22/152) or severe intracranial hemorrhage (2.63%, 4/152), and 126 rats were included in the nine orthogonal test subgroups (N_i_ = 14, i = 1, 2 to 9). Minor side effects, such as shivering, were observed in 5.56% (7/126) after anesthetic resuscitation. No arrhythmia, infection, or shock was found in all the subgroups. No statistical differences were found in the nine subgroups' mortality and complications. Animals' body temperature, heart rate, respiratory rate, and blood oxygen saturation were detected prior to and post-IAH intervention (Supplementary Table S1). Core temperature decreased by 0.2–0.5 °C in each IAH subgroup after cold NS perfusion. Blood oxygen saturation decreased by 1.5–5.4% but remained above 90%. No significant pathological abnormal changes were found in heart rate, pulse, or respiratory rate. Blood routine tests and biochemical indicators, including blood cell count, hemoglobin concentration, hematocrit, albumin, cholesterol, electrolytes, blood glucose, myocardial enzymes, liver, and kidney function, were performed prior to and post-IAH (Supplementary Tables S2, S3). After IAH intervention, the number of red blood cells, hemoglobin concentration, hematocrit, and serum calcium were significantly decreased, while blood glucose, cholesterol, alkaline phosphates, alanine aminotransferase, aspartate aminotransferase, and creatine kinase increased slightly. There were no significant changes in albumin, urea nitrogen, and creatine levels. All indicators were no significant pathological abnormality according to the previously reported normal references^26^.

### Changes of T_b_, T_core_, and T_jvb_

#### Dynamic changes of T_b_, T_core_, and T_jvb_

**Figure 2:**
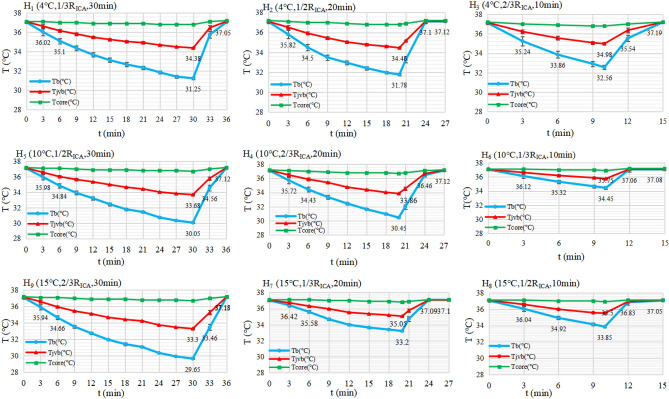
Temperature-time curves of different local temperatures stratified to H_1_-H_9_ subgroups in MCAO/R rats during IAH. Temperatures (T_core_, T_b_, and T_jvb_) were detected on the time-points every three minutes from the start, and some additional observatory time-points such as the 10, 20 min. Nine plots of all subgroups were arrayed in columns according to the continuous infusion time. The temporal changes of different local temperatures during IAH were shown with three curves: T_b_ (blue line), T_jvb_ (red line), and T_core_ (green line), respectively. As shown, T_b_ decreased rapidly at first and then gradually reached the minimum until stopping the infusion, then recovered to the baseline level within about 5 min. T_jvb_ had a similar tendency as T_b_ and was closely correlated with T_jvb_ in every subgroup (R_i_ > or = 0.996, *P*_i_ < 0.001, i = 1,2…9, by Pearson correlation). The reductions of T_core_ were all within 1.0 °C in each subgroup. Notes: H_1_: 4 °C,1/3 R_ICA_, 30 min; H_2_: 4 °C, 1/2 R_ICA_, 20 min; H_3_: 4 °C, 2/3 R_ICA_, 10 min; H_4_: 10 °C, 2/3 R_ICA_, 20 min; H_5_: 10 °C,1/2 R_ICA_, 30 min; H_6_: 10 °C, 1/3 R_ICA_, 10 min; H_7_: 15 °C, 1/3 R_ICA_, 20 min; H_8_: 15 °C,1/2 R_ICA_,10 min; H_9_: 15 °C, 2/3 R_ICA_, 30 min; R_ICA_, the blood flow rate of the internal carotid artery (ICA) per minute; IAH, Intra-arterial therapeutic hypothermia; T, temperature; t, time; T_b_, ischemic brain tissue local temperature (the solid line in blue); T_jvb_, ipsilateral jugular venous bulb temperature (the solid line in red); T_core_, the core temperature (that is the anus temperature) (the solid green line); MCAO/R, middle cerebral artery occlusion, and reperfusion; TH: therapeutic hypothermia.

The Temperature -Time curves of T_b_, T_core_, and T_jvb_ during IAH for each subgroup are shown in Fig. 2. During IAH intervention, T_b_ (blue line) could be decreased to ≤ 35 °C in several min, then gradually reach the bottom of around 30 °C in every subgroup. When stopping the infusion, it would be recovered to the baseline level within 5 min. T_jvb_ changed with a similar trend as T_b_ but with a lower magnitude. T_b_ was closely correlated with T_jvb_ in every subgroup (R_i_ ≧ 0.996, *P*_*i*_ < 0.001, i = 1, 2 to 9, by Pearson correlation), which indicated T_jvb_ could estimate T_b_ in actual practice. T_core_ was reduced by 1.0 °C, suggesting that IAH has little effect on the core body temperature.

#### Analysis for T_b_-related indexes

The T_b_-related indexes during IAH for each subgroup are listed in Table 2. The Time of T_b-35 °C_ of the H_3_ subgroup (4 °C, 2/3 R_ICA_, 10 min) was 3.43 ± 0.53 min, which was significantly lower than any other subgroups (*P* < 0.001, by Dunnett's *t*-test)^22^. The results indicated that the lower perfusate temperatures and the faster perfusion flow rate might cool faster. The time of TH being kept below 35 °C was regarded as the valid time of TH. The H_9_ subgroup (15 °C, 2/3 R_ICA_, 30 min) had a longer time of TH (28.71 ± 0.49 min) and longer rewarming time than any other subgroups (*P* < 0.001, by Dunnett's t-test), which indicated that the faster infusion rate and the longer perfusion time, the longer time of TH would be.

**Table 2:** Effects of IAH on the related indexes according to the nine orthogonal test subgroups (Mean ± SD).

Indexes	H_1_ (4 °C, 1/3 R_ICA_, 30 min, 7.5 ml)	H_2_ (4 °C, 1/2 R_ICA_, 20 min, 7.5 ml)	H_3_ (4 °C, 2/3 R_ICA_, 10 min, 5.0 ml)	H_4_(10 °C, 2/3R_ICA_, 20 min, 10.0 ml)	H_5_(10 °C,1/2 R_ICA_, 30 min, 11.3 ml)	H_6_(10 °C, 1/3R_ICA_, 10 min, 2.5 ml)	H_7_(15 °C,1/3 R_ICA_, 20 min, 5.0 ml)	H_8_(15 °C,1/2R_ICA_, 10 min, 11.3 ml)	H_9_(15 °C,2/3 R_ICA_, 30 min,15.0 ml)
The T_b_-related indexes									
Time of T_b-35_ °C (min, n = 7)	6.14 ± 0.38**	4.71 ± 0.49**	3.43 ± 0.53^R^	4.86 ± 0.38**	5.71 ± 0.49**	7.43 ± 0.53**	7.71 ± 0.49**	5.86 ± 0.38**	4.71 ± 0.49**
T_b-min_ ( °C, n = 7)	31.25 ± 0.08**	31.78 ± 0.09**	32.56 ± 0.16**	30.45 ± 0.09**	30.05 ± 0.05**	34.45 ± 0.13**	33.20 ± 0.06**	33.85 ± 0.08**	29.65 ± 0.06^R^
Time of TH (min, n = 7)	26.29 ± 0.49**	17.57 ± 0.53**	7.71 ± 0.49**	18.57 ± 0.53**	27.71 ± 0.49**	3.29 ± 0.49**	13.43 ± 0.53**	4.43 ± 0.53**	28.71 ± 0.49^R^
Rewarming time (min, n = 7)	3.86 ± 0.38**	3.43 ± 0.53**	2.86 ± 0.38**	4.43 ± 0.53^NS^	4.57 ± 0.53^NS^	1.71 ± 0.49**	2.57 ± 0.53**	2.14 ± 0.38**	4.86 ± 0.38^R^
The outcome indexes									
Infarction volume (%, n = 7)	27.05 ± 1.73**	23.96 ± 2.54^NS^	24.14 ± 1.68^NS^	25.84 ± 1.42*	28.76 ± 1.64**	33.42 ± 2.51**	32.78 ± 2.02**	31.86 ± 1.86**	23.58 ± 2.10^R^
Cerebral water content (%, n = 7)	81.51 ± 0.21**	80.42 ± 0.18^NS^	80.53 ± 0.17*	80.77 ± 0.14**	81.91 ± 0.18**	82.73 ± 0.20**	82.61 ± 0.17**	82.51 ± 0.18**	80.32 ± 0.18^R^
mNSS ( n = 7)	8.43 ± 1.40^NS^	7.86 ± 1.35^NS^	7.71 ± 1.50^NS^	8.00 ± 1.41^NS^	8.43 ± 1.62^NS^	10.86 ± 2.12**	9.86 ± 1.86**	10.00 ± 1.63**	7.57 ± 1.72^R^

IAH, intra-arterial cerebral hypothermia; SD, standard deviation; T_b_, local brain tissue temperature; Time of T_b-35_ °C, Time of T_b_ initially reaching below 35 °C; T_b-min_, the minimum of T_b_; Rewarming time, Time of T_b_ rewarming to normal body temperature. MCAO/R, middle cerebral artery Occlusion, and Reperfusion; mNSS, modified Neurological Severity Score; H_1_: 4 °C,1/3 R_ICA_, 30 min; H_2_: 4 °C, 1/2 R_ICA_, 20 min; H_3_: 4 °C, 2/3 R_ICA_, 10 min; H_4_: 10 °C, 2/3 R_ICA_, 20 min; H_5_: 10 °C,1/2 R_ICA_, 30 min; H_6_: 10 °C, 1/3 R_ICA_, 10 min; H_7_: 15 °C, 1/3 R_ICA_, 20 min; H_8_: 15 °C,1/2 R_ICA_,10 min; H_9_: 15 °C, 2/3 R_ICA_, 30 min; R_ICA_, blood flow Rate of the internal carotid artery (ICA).

*P < 0.05, **P < 0.01, ^NS^no significance, ^R^the Reference subgroup, by the Dunnett test.

### Effects of IAH on the outcome-related indexes

#### Cerebral infarction volume

**Figure 3:**
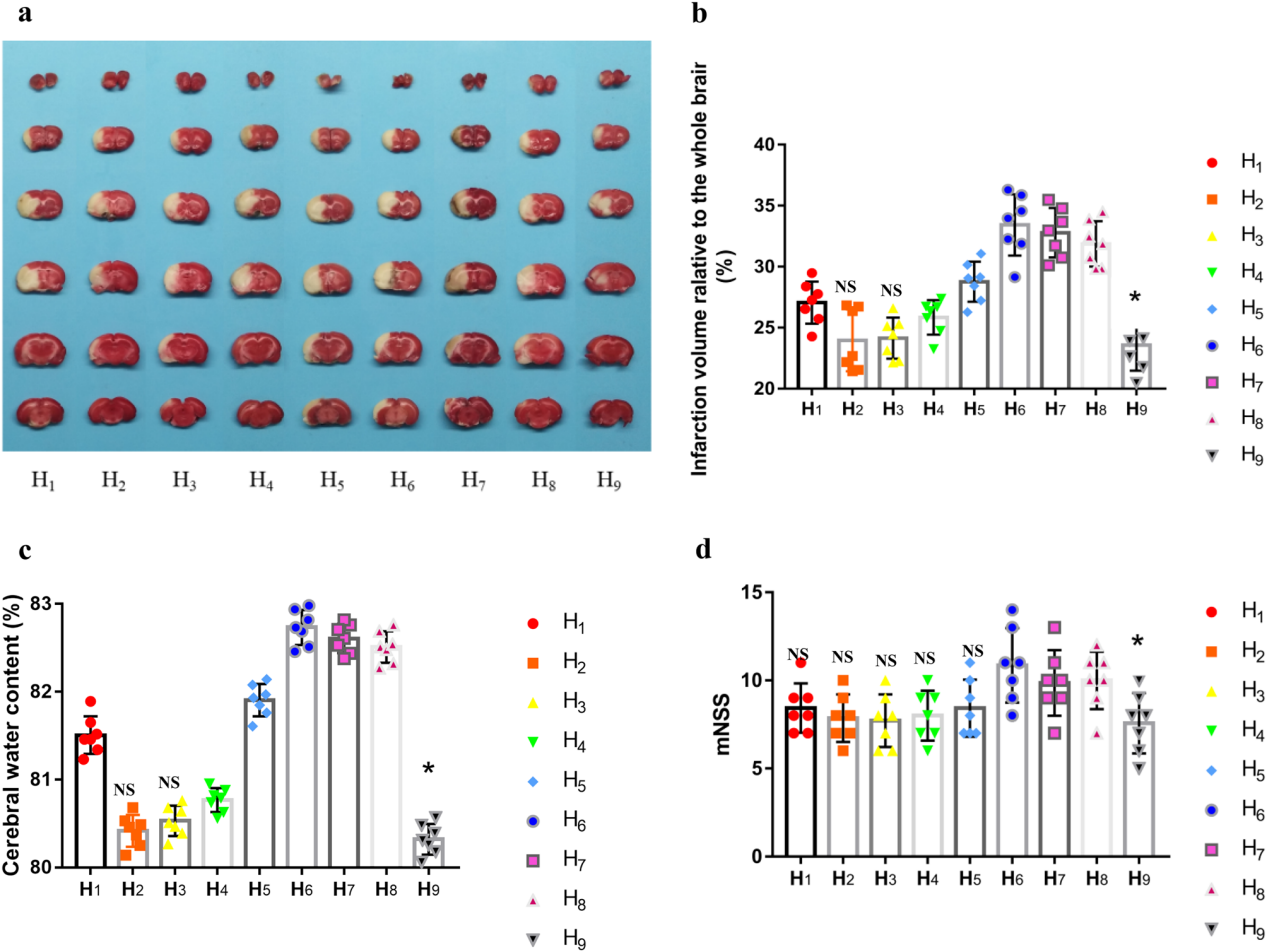
Effects of IAH on the MCAO/R rats in the H_1_–H_9_ subgroups. (**a**) The representative images for TTC staining of brain tissue. (**b**) Ratios of the cerebral infarction volume to total brain tissue volume in TTC staining. The red parts of the brain tissue reflected normal tissue; the white parts represented cerebral infarction tissue. The ratio of infarct volume in each subgroup ranged from 23.58 to 33.42%. The H_9_ subgroup had the smallest proportion of cerebral infarction volume (23.58 ± 2.10%) but had no statistical differences compared with the H_2_ and H_3_ subgroup, respectively (Ni = 7, *P*_*i*_ > 0.05, i = 2, 3, By Dunnett test; * the reference subgroup). (**c**) The cerebral water content in each IAH subgroup. The H_9_ subgroup had the better cerebral water content (80.32 ± 0.18%) but had no significant difference with that of the H_2_ subgroup, respectively (Ni = 7, *P*_*i*_ > 0.05, i = 2, By Dunnett test; * the reference subgroup). (**d**) The neurological function (mNSS) in each IAH subgroup. The H_9_ subgroup had the better mNSS (7.57 ± 1.72) but had no significant difference with that of the H_1_-H_5_ subgroup, respectively ( Ni = 7, *P*_*i*_ > 0.05, i = 1–5, By Dunnett test; * the reference subgroup). Notes: IAH: Intra-arterial therapeutic hypothermia; T: temperature; t; time; T_b_, ischemic brain tissue local temperature (the solid line in blue); MCAO/R: middle cerebral artery occlusion and reperfusion; TTC: 2,3, 5-Triphenyltetrazolium chloride; mNSS: modified Neurological Severity Score; TH: therapeutic hypothermia; H_1_: 4 °C,1/3 R_ICA_, 30 min; H_2_: 4 °C, 1/2 R_ICA_, 20 min; H_3_: 4 °C, 2/3 R_ICA_, 10 min; H_4_: 10 °C, 2/3 R_ICA_, 20 min; H_5_: 10 °C,1/2 R_ICA_, 30 min; H_6_: 10 °C, 1/3 R_ICA_, 10 min; H_7_:15 °C, 1/3 R_ICA_, 20 min; H_8_:15 °C,1/2 R_ICA_,10 min; H_9_: 15 °C, 2/3 R_ICA_, 30 min; R_ICA_: blood flow rate of the internal carotid artery (ICA) per minute.

The cerebral infarction volume was quantified by TTC (n_i_ = 7, i = 1–9) 24 h after intervention (Fig. 3a, b). The results showed a well-defined ischemic area (white part) delimited by normal brain tissue (red part) that differed between subgroups.The results showed a well-defined ischemic area (white part) delimited by normal brain tissue (red part) that differed between subgroups.The results showed a well-defined ischemic area (white part) delimited by normal brain tissue (red part) that differed between subgroups. The ratio of infarct volume in each subgroup ranged from 23.58 to 33.42%. The H_9_ subgroup (15 °C, 2/3 R_ICA_, 30 min) had the smallest proportion of cerebral infarction(23.58 ± 2.10%), which was significantly smaller than that of the H_1_ and H_4_-H_8_ subgroups (*P* < 0.05, by Dunnett's *t*-test), but no statistical difference compared with the H_2_ and H_3_ subgroups.

Effects of IAH, stratified by the three infusion factors and their levels, on cerebral infarction volume were listed in Table 3. For factor A, the mean of Level1, Level2, and Level3 were 25.05 ± 0.45%, 29.34 ± 0.45%, and 29.41 ± 0.45%, respectively. The better level was directly identified as A1, according to the mean range of Level 3 and Level 1 (4.29%). For the factor B, the mean of Level1, Level2, Level3 were 31.08 ± 0.45%, 28.19 ± 0.45% and 24.52 ± 0.45%, respectively, so the better level was B3, according to the mean range of Level3 and Level1(6.56%). For factor C, the mean of Level1, Level2, and Level3 were 29.81 ± 0.45%, 27.53 ± 0.45%, and 26.46 ± 0.45%, respectively, so the better level was C3 according to the mean range of Level3 and Level2 (3.35%). Factor B might be the greatest influence factor on the protective effects of IAH, and an optimal combination of A1B3C3 was postulated based on the range values.

**Table 3:** Effects of IAH on the outcome indexes for MCAO/R rats stratified to the infusion factors and their levels.

LEVEL _i_	Infraction volume (TTC, %)			Cerebral water content (%)			mNSS		
(Ni = 21, i = 1–3)	Factor A	Factor B	Factor C	Factor A	Factor B	Factor C	Factor A	Factor B	Factor C
L_1_ (M ± SD)	***25.05*** ± ***0.45***	31.08 ± 0.45	29.81 ± 0.45	***80.82*** ± ***0.07***	82.28 ± 0.07	81.93 ± 0.07	***8.00*** ± ***0.35***	9.71 ± 0.35	9.52 ± 0.35
L_2_ (M ± SD)	29.34 ± 0.45	28.19 ± 0.45	27.53 ± 0.45	81.80 ± 0.07	81.61 ± 0.07	81.26 ± 0.07	9.10 ± 0.35	8.76 ± 0.35	8.57 ± 0.35
L_3_ (M ± SD)	29.41 ± 0.45	***24.52*** ± ***0.45***	***26.46*** ± ***0.45***	81.81 ± 0.07	***80.54*** ± ***0.07***	***81.25*** ± ***0.07***	9.14 ± 0.35	***7.76*** ± ***0.35***	***8.14*** ± ***0.35***
Range^(1)^	4.29	6.56	3.35	0.99	1.74	0.68	1.14	1.95	1.38
Better Level^(2)^	A1	B3	C3	A1	B3	C3	A1	B3	C3
Factor Rank	BAC			BAC			BCA		
Better combination^(3)^	A1B3C3			A1B3C3			A1B3C3		
Multivariate analysis by ANOVA of the orthogonal test									
F values	30.460	52.881	14.268	75.775	179.537	34.857	3.382	7.711	4.042
* P*	< 0.001	< 0.001	< 0.001	< 0.001	< 0.001	< 0.001	0.041	0.001	0.023
Factor rank^(4)^	BAC			BAC			BCA		
Multicomparison between levels by LSD									
*P*_*12*_ (L_1_ vs L_2_)	< 0.001	< 0.001	0.001	< 0.001	< 0.001	< 0.001	0.032	0.061	0.061
*P*_*13*_ (L_1_ vs L_3_)	< 0.001	< 0.001	< 0.001	< 0.001	< 0.001	< 0.001	0.025	< 0.001	0.007
*P*_*23*_ (L_2_ vs L_3_)	0.916	< 0.001	0.102	0.906	< 0.001	0.838	0.924	0.049	0.392
Better level^(5)^	A1	B3	C2 & C3	A1	B3	C2 & C3	A1	B3	C2 & C3
Better combinations^(6)^	A1B3C3 or A1B3C2			A1B3C2 or A1B3C3			A1B3C3 or A1B3C2		

Statistical estimated preferred combinations: A1B3C2 (4 °C, 2/3R_ICA_, 20 min) or A1B3C3 (4 °C, 2/3R_ICA_, 30 min).

The preferred optimal conditions: *A1B3C2 (4 °C, 2/3R*_*ICA*_*, 20 min)*(7).

Significant values are in bolditalics.

IAH, intra-arterial cerebral hypothermia; MCAO/R, middle cerebral artery Occlusion and Reperfusion; TTC, 2, 3, 5-triphenyl tetrazolium chloride; mNSS, modified Neurological Severity Score; Ni, the number of subjects in a certain level of the factors; ANOVA, Analysis of Variance; R_ICA_, blood flow rate of the internal carotid artery (ICA); SD, standard deviation; LSD, a least significant difference. (1) the range between the max and min means among the three levels; (2) the level of every factor with a minimized mean according to the clinical implication of the index; (3) the combination of the factor and its better level with a better index; (4) Factor rank according to the F value of multivariate analysis by ANOVA of the orthogonal test; (5) the level with a minimize mean according to the multicomparison analysis; (6) the combination of the factor and its better level according to statistical analysis; (7) the preferred combination with a less total infusion volume than the other one.

Multivariate analysis by ANOVA of the orthogonal tests showed that perfusate temperature (F = 30.460, *P* < 0.001), Perfusate rate (F = 52.881, *P* < 0.001), and infusion duration (F = 14.268, *P* < 0.001) were respectively independent factors on the cerebral infarction volume. The order of action strength should be the perfusate rate, temperature, and infusion duration according to the respective F values. Further analysis of multicomparison showed that there were significant differences between Level1 and Level2 or Level3 for factor A (*P* < 0.001), any two of the three levels for factor B (*P* < 0.001), and Level1 and Level2 for factor C (*P* = 0.001), but no significant difference was found between L3 and Level 2 for factor C (*P* = 0.102). These results indicated the better levels were A1, B3, C2/C3, and the estimated optimal combination was A1B3C3(4 °C, 2/3 R_ICA_, 30 min) or A1B3C2(4 °C, 2/3 R_ICA_, 20 min).

#### mNSS

Subjects were assessed for mNSS in every subgroup (n_i_ = 7, i = 1–9) 72 h after intervention (Table 2, Fig. 3d). The H_9_ (15 °C,2/3 R_ICA_, 30 min) subgroup had the best mNSS (7.57 ± 1.72), which was a statistically significant difference compared with that of the H_6_, H_7_, and H_8_ subgroup, respectively (*P* < 0.01, by Dunnett's* t*-test), but had no significant difference with that of the H_1_- H_5_ subgroup, respectively (*P* > 0.05). These results indicated that the H_9_ subgroup (15 °C, 2/3 R_ICA_, 30 min) might be the optimal conditions of IAH.

The analysis of mNSS, stratified by the three infusion factors and their levels were listed in Table 3. For factor A, the mean of Level 1, Level 2, and Level 3 was 8.00 ± 0.35, 9.10 ± 0.35, and 9.14 ± 0.35, respectively. The better level was directly identified as A1, according to the mean range of Level 3 and Level 1(1.14). For factor B, the mean of Level1, Level2, and Level3 were 9.71 ± 0.35, 8.76 ± 0.35 and 7.76 ± 0.35, respectively, so the better level was B3 according to the mean range of Level3 and Level1(1.95). For factor C, the mean of Level1, Level2, and Level3 were 9.52 ± 0.35, 8.57 ± 0.35 and 8.14 ± 0.35, respectively, so the better level was C3 according to the mean range of Level3 and Level1 (1.38). Factor B might be the greatest influence factor on mNSS. These results also indicated that the combination of A1B3C3 is the optimal condition of IAH.

Multivariate analysis for mNSS was performed by ANOVA of the orthogonal tests (Table 3). Perfusate temperature (F = 3.382, *P* = 0.041), Perfusate rate (F = 7.711, *P* = 0.001) and infusion duration(F = 4.042, *P* = *0.023*) were respectively independent factors on mNSS. The order of action strength should be the perfusate rate, infusion duration, and perfusate temperature according to the respective F values. Further analysis of multicomparison showed that there were significant differences between Level1 and Level2 or Level3 for factor A (*P* = 0.041), any two of the three levels for factor B (*P* < 0.001), and Level1 and Level2 for factor C (*P* = 0.023), but no significant difference was found between L3 and Level2 for factor C (*P* = 0.392). These results demonstrated the better levels were A1, B3, C2/C3, and the estimated optimal combination was A1B3C3(4 °C, 2/3 R_ICA_, 30 min) or A1B3C2 (4 °C, 2/3 R_ICA_, 20 min).

#### Cerebral water content

After the evaluation of mNSS, the subjects were used for cerebral water content in every subgroup (n_i_ = 7, i = 1–9) 72 h after intervention (Table 2, Fig. 3c). The total water content of brain tissue was used as a surrogate measurement for cerebral edema. The H_9_ (15 °C, 2/3 R_ICA_, 30 min) subgroup had the least cerebral water content (80.32 ± 0.18%), which was a statistically significant difference compared with that of the H_1_, and H_3–8_ subgroup, respectively (*P* < 0.01, by Dunnett's *t*-test), but had no significant difference with that of the H_2_ subgroup, respectively (*P* > 0.05). These results indicated that the H9 subgroup (15 °C, 2/3 RICA, 30 min) are optimal conditions of IAH.

Analysis of cerebral water content, stratified by the three infusion factors and their levels, were listed in Table 3. For factor A, the mean of Level1, Level2, and Level3 were 80.82 ± 0.07%, 81.80 ± 0.07%, and 81.81 ± 0.07%, respectively. The better level was directly identified as A1, according to the mean range of Level 3 and Level 1(0.99%). For factor B, the mean of Level1, Level2, and Level3 were 82.28 ± 0.07%, 81.61 ± 0.07%, and 80.54 ± 0.07%, respectively, so the better level was B3 according to the mean range of Level3 and Level1(1.74%). For factor C, the mean of Level1, Level2, and Level3 were 81.93 ± 0.07%, 81.26 ± 0.07%, and 81.25 ± 0.07%, respectively, so the better level was C3 according to the mean range of Level3 and Level1 (0.68%). Factor B might be the greatest influence factor on cerebral water content. These results also indicated that the combination of A1B3C3 is the optimal condition of IAH.

Multivariate analysis for cerebral water content was performed by ANOVA of the orthogonal tests (Table 3). Perfusate temperature (F = 75.775, *P* < 0.001), Perfusate rate (F = 179.537, *P* < 0.001), and infusion duration (F = 34.857, *P* < 0.001) were respectively independent factors on cerebral water content. The order of action strength should be the perfusate rate, temperature, and infusion duration according to the respective F values. Further analysis of multicomparison showed that there were significant differences between Level1 and Level2 or Level3 for factor A (*P* < 0.001), any two of the three levels for factor B (*P* < 0.001), and Level1 and Level2 for factor C (*P* < 0.001), but no significant difference was found between L3 and Level2 for factor C (*P* = 0.838). These results demonstrated the better levels were A1, B3, C2/C3, and the estimated optimal combination was A1B3C3(4 °C, 2/3 R_ICA_, 30 min) or A1B3C2(4 °C, 2/3 R_ICA_, 20 min).

According to the above analysis for cerebral infarction volume, cerebral water content, and mNSS, the estimated optimal conditions of IAH should be 4 °C, 2/3 R_ICA_, 20/30 min. Considering there were no significant differences in cerebral infarct volume, cerebral water content, and mNSS between the 30 min and 20 min infusion, less total perfusate volume may reduce the overload risk of body humoral for model rats. Thus the optimal theoretical scheme of IAH was supposed to be 4 °C, 2 / 3 R_ICA,_ and 20 min.

## Discussion

TH is a promising neuroprotective approach that can play a role in the ischemic cascade targeting multiple links of the pathophysiological process. IAH is safer than systemic hypothermia and is potentially effective for AIS^20,27–30^, which may be carried on with a dedicated closed-loop tube or intracarotid cold infusion (ICCI). Cattaneo G et al. reported a special intracarotid cooling catheter conceived for fast and selective brain cooling during endovascular thrombectomy in an ovine stroke model^17^. Wang Y and his colleagues introduced a dedicated infusion system for continuous pre- to post-reperfusion ICCI in rat stroke models^18^. Intracarotid infusion of cold saline had been suggested as a practicable and effective cooling method and was investigated in small animal experiments^15^. These studies indicated that IAH was promising in clinical transformation, but the infusion conditions and related influence factors were generally different and empirically determined. In this study, we systematically explored the conditions of ICCI in an MCAO/R rat model, especially some factors that might be important for perfusion efficacy and safety, such as perfusion temperature, infusion rate, and infusion duration. We found that the three factors were all independent influence factors of the IAH effect and demonstrated that the optimized IAH scheme of 4 °C cold NS, 2/3 R_ICA_ infusion rate for 20 min, could rapidly achieve TH for about 20 min, and significantly reduced the infarct volume and cerebral water content, partially improved neurological function of the model rats.

The primary safety concerns for IAH were the possibility of increased cerebral edema and the tolerance of the subjects to fluid volume expansion caused by the injection of a large volume of hypothermic saline within a short period, and secondly for the effects of local brain cooling on clinical endpoints such as neurological function and neuropathology. Perfusate temperature, perfusion flow rate, and saline infusion duration were considered the three key factors of IAH. The appropriate infusion flow rate should consider the diameter, ICA (RICA) blood flow rate, and systemic blood volume. The normal ICA flow velocity (V_ICA_)of adult SD rats (240–265 g) is about 50 cm/s^21^, and the blood flow rate (R_ICA_) could be estimated to be about 0.75–2.0 mL/min^22^. Considering both the rat blood volume and the total infusion volume, the lower R_ICA_ value of 0.75 mL/min was taken as the reference. This study set the max infusion rate as 0.5 ml/min, similar to a previous study with 0.5–0.7 ml/h^18^.

Additionally, the effects of IAH are generally closely related to the temperature of cold perfusate. The perfusate temperature and continuous time may directly determine the decreasing speed and extent of local temperature and affect the neuroprotective of IAH. The temperature of the mixture (mixing blood and saline) on the outlet of the catheter (T_CP_) is related to R_ICA_ and the temperature of the cold perfusate (T_0_), which can be described with formula^31^:

$$p = \frac{{ICA\;Blood \;Flow \cdot 37 + Saline\;Flow \cdot T_{0} }}{ICA\;Blood\;Flow + Saline\;Flow}$$

When T_0_ is 15 °C, and the perfusion flow rate is 1/3 R_ICA_, T_CP_ is 31.55 °C, which may have a poor cooling effect on the local brain. Therefore, the proposed perfusion flow rate should be ε1/3 R_ICA_. However, when the perfusion flow rate is 2/3 R_ICA_, the maximum infusion volume can be close to the whole blood volume in 30 min, and the animal may not tolerate it, so the perfusion flow rate should be ≤  δ 2/3 R_ICA_. Factors for infusion duration should be considered, including the feasibility of the experimental procedure, the animal's tolerance to total fluid volume, and the duration of hypothermia. In clinical intra-arterial thrombolysis or mechanical recanalization, it is challenging to carry out more than a few h for IAH. Konstas et al. considered prolonged hypothermia unnecessary, as a too-long IAH may affect the tissue's normal oxygen supply and nutrient requirements, exacerbating systemic side effects and complications^31^.

Therefore, we conduct an experiment design of 3 factors with 3 levels: perfusate temperature with 4, 10, and 15 °C, perfusion flow rate at 1/3, 1/2, and 2/3 R_ICA_, perfusion duration for 10, 20, or 30 min, with a total perfusate volume that does not exceed 15 ml (Table 1). Except for some statistically varied indicators (e. g., PaO_2_, Hb, and HCT), no significant abnormal changes were observed in the vital signs, blood routine, and biochemical indicators before and after perfusion in all subgroups (Supplementary Tables S1–S3)^26^. The model rats tolerated ICCH well under these conditions without severe complications and side effects. Only a few hypothermia-related side effects, such as shivering, were observed, which may be related to slight changes in T_core_ and a relatively short observation time. Although twenty-two rats died during the intervention, most were supposed to be dead from the surgery operation but not ICCH. Therefore, the predesigned scheme of IAH was safe and feasible for MCAO/R rats.

In this study, we demonstrated that perfusate temperature, infusion flow rate, and infusion duration are all independent predictors of infarction volume, cerebral water content, and mNSS, and perfusion flow rate showed the greatest impact, followed by perfusate temperature. The greater the perfusion speed, the lower the perfusion temperature, and the longer the perfusion duration, the better the cooling effect of IAH. It is possible that, combined with lower perfusion temperature for a longer duration, a faster perfusion rate can reach TH faster in the local brain, and maintain for a longer time, thus playing more effective neuroprotection. The preliminarily estimated optimal perfusion condition in MCAO/R rats was 4 °C, 2/3 R_ICA_, for 20 min or 30 min according to the index of cerebral infarction volume, cerebral water content, and mNSS (Tables 2, 3; Figs. 2, 3). The mNSS showed no significant difference in cerebral water content and infarct volume between either level, which may be related to a relatively short-term observation and the insufficient recovery of neurological deficit. For factor C, the three actual outcome indexes of Level 3 were better than those of Level 2, but no significant differences were found between levels (Table 3). Surprisingly, in the subgroup with higher perfusion volume, it may be explained by effective hypothermia, protecting the blood–brain barrier and reducing edema, which was similar to clinical findings that cerebral edema was decreased after reperfusion in anterior circulation infarction^32^. Nevertheless, in theory, less total perfusion volume would be safer in case of the same similar efficacy. Therefore, according to this study setting, the optimal IAH condition was supposed to be 4 °C, 2/3 R_ICA_, for 20 min.

Detection and monitoring the local temperature of ischemic brain tissue are technically challenging, especially in clinical practice. Here, we monitored T_b_ with a traumatic method not translatable to clinical practices. During IAH, T_b_ and T_jvb_ decreased rapidly during the early min and then continuously slower until the end of perfusion treatment. In this study, changes in core temperature were insignificant (Fig. 2), which may be related to a low incidence of hypothermia-related physiological-biochemical influences and side effects (Supplementary Tables S2, S3, S4). Konstas firstly reported the mathematical model of T_b_ and T_jvb_, and then Neimark reported a forward mathematical model, where the brain temperature could be predicted based on the infused saline temperature, and a reverse model, where the brain temperature is inferred based on jugular venous bulb temperature (T_jvb_)^32,33^. Corrected by T_jvb_, the mathematical model could be used as a noninvasive estimation for transient and steady-state brain tissue temperature changes.

Consequently, even if the local tissue temperature of the human brain can not be obtained directly, it can be indirectly postulated by the reasonable degree of actual measurement and theoretical model. Herein, we observed a significant correlation between T_b_ and ipsilateral T_jvb_ and an experimentally validated Neimark et al*.* mathematical model where Tjvb inferred the brain temperature following IAH^33^. Accordingly, in clinical practices, temperature changes in ischemic brain tissue can be predicted by the decrease in T_jvb_ that can be easily measured by less evasive procedures, which may guide the adjustment of perfusion parameters for IAH, increase therapeutic effectiveness, and decrease side effects and complications. Moreover, this highlights the importance of engineering improvements in hypothermia instruments, where an automatic temperature control function based on T_jvb_ would ease IAH clinical translation.

Although the optimized conditions were explored for IAH, this study has some limitations. Firstly, the conclusions drawn from rodents always need further rigorous adjustment and verification before translation into clinical practice, where other pivotal independent predictors not tested in this study, such as IAH's time window, optimal target temperature, and rewarming speed, can play a significant impact on the herein tested settings. Secondly, the tested conditions were selected according to previous references and the rats' anatomical-physiological characteristics, which does not mean all possible best conditions, such as a lower perfusate temperature of 0 °C, might be better. Thirdly, some important parameters such as blood pressure, intracranial pressure, and the integration of brain blood barrier were not investigated for limited study time and conditions, which might be further studied with the optimized infusion condition. Finally, this study did not investigate the potential protective mechanisms of IAH. Several studies have suggested a neuroprotective effect of vascular washing, where improving blood circulation at the cerebral lesion can reduce the accumulation of local harmful pathological products^34^.

## Conclusion

In conclusion, IAH is safe and feasible in an MCAO/R rat model. In this model, 4 °C NS perfusion at 2/3 R_ICA_ for 20 min was optimal for decreasing cerebral infarction volume and cerebral water content and improving neurological deficits. Furthermore, we show that T_jvb_ is significantly correlated to T_b_ and can be used as a surrogate measurement to predict local ischemic brain tissue cooling. Altogether, these findings provide uniform settings for IAH therapy in rat models and will guide future mechanistic studies of IAH neuroprotection, which is crucial for therapeutic translation to the clinic. It seems that IAH might be safer and more feasible than systemic hypothermia in clinical thrombectomy for LVO-AIS.

## Supplementary Information

Supplementary Information.

## Data Availability

The datasets generated during and/or analyzed during the current study are available from the corresponding author upon reasonable request.
