# Supplementary material for: Optimizing intra-arterial hypothermia scheme for acute ischemic stroke in an MCAO/R rat model
Source: Sci Rep. 2023 Jun 13;13:9566. doi: 10.1038/s41598-023-35824-y (PMC10264431; doi:10.1038/s41598-023-35824-y)
Supplement: Supplementary file 1 — Supplementary Information. [file 41598_2023_35824_MOESM1_ESM.docx]

| **Supplementary Table 1. The number of rats, the incidence of complications, and the survival rate were stratified into subgroups.** | | | | | | | | | |
| --- | --- | --- | --- | --- | --- | --- | --- | --- | --- |
| **indexes** | **H_1_**  (4℃,1/3R_ICA_, 30 min) | **H_2_**  (4℃,1/2R_ICA_, 20 min) | **H_3_**  (4℃,2/3R_ICA_, 10 min) | **H_4_**  (10℃,2/3R_ICA_, 20 min) | **H_5_**  (10℃, 1/2 R_ICA_, 30 min) | **H_6_**  (10℃, 1/3R_ICA_, 10 min) | **H_7_**  (15℃, 1/3 R_ICA_, 20 min) | **H_8_**  (15℃,1/2R_ICA_, 10 min) | **H_9_**  (15℃,2/3R_ICA_, 30 min) |
| Number of used | 18 | 17 | 16 | 18 | 18 | 16 | 16 | 16 | 17 |
| Number of included (n, %) | 14 (77.8%) | 14 (82.4%) | 14 (87.5%) | 14 (77.8%) | 14 (77.8%) | 14(87.5%) | 14(87.5%) | 14(87.5%) | 14 (82.4%) |
| Number of excluded (n, %) | 4 (22.2%) | 3 (17.6%) | 2 (12.5%) | 4 (22.2%) | 4 (22.2%) | 2 (12.5%) | 2 (12.5%) | 2 (12.5%) | 3 (17.6%) |
| Died from surgery (n, %) | 3 (16.7%) | 3 (17.6%) | 2 (12.5%) | 3 (16.7%) | 3 (16.7%) | 2 (12.5%) | 2 (12.5%) | 2 (12.5%) | 2 (11.8%) |
| Intracranial hemorrhage (n, %) | 1 (5.6%) | 0 (0%) | 0 (0%) | 1 (5.6% ) | 1 (5.6%) | 0 (0%) | 0 (0%) | 0 (0%) | 1 (5.9%) |
| Survival rate (n, %) | 15(83.3% ) | 14(82.4%) | 14(87.5% ) | 15(83.3%) | 15 (83.3%) | 14(87.5%) | 14 (87.5%) | 14 (87.5%) | 15(88.2%) |
| Shiver (n, %) | 1 (5.6%) | 1 (5.9%) | 1 (6.25%) | 1 (5.6%) | 1 (5.6%) | 0 (0%) | 0 (0) | 1 (6.25%) | 1 (5.9%) |

***Notes:*** R_ICA_: blood flow rate of the internal carotid artery (ICA) per minute. H_1_: 1/3 R_ICA_, 30 min, 4 ℃; H_2_: 1/2 R_ICA_, 20 min, 4 ℃; H_3:_ 2/3 R_ICA_, 10 min, 4 ℃; H_4:_ 2/3 R_ICA_, 20 min, 10 ℃; H_5:_ 1/2 R_ICA_, 30 min, 10 ℃; H_6:_ 1/3 R_ICA_, 10 min, 10 ℃; H_7:_ 1/3 R_ICA_, 20 min, 15 ℃; H_8:_ 1/2 R_ICA_, 10 min, 15 ℃; H_9:_ 2/3 R_ICA_, 30 min, 15 ℃.

| **Supplementary Table 2. The effects of IAH on vital signs of MCAO/R model rats in each subgroup.** | | | | | | | | | | |
| --- | --- | --- | --- | --- | --- | --- | --- | --- | --- | --- |
| **Indexes** | **Detection time** | **H_1_** | **H_2_** | **H_3_** | **H_4_** | **H_5_** | **H_6_** | **H_7_** | **H_8_** | **H_9_** |
| T  (℃) | Before | 37.1±0.2 | 37.2±0.2 | 37.2±0.2 | 37.2±0.2 | 37.2±0.2 | 37.1±0.2 | 37.1±0.2 | 37.1±0.2 | 37.2±0.2 |
|  | After | 36.8 ±0.2* | 36.8±0.2** | 36.8±0.2** | 36.7±0.2** | 36.7±0.2** | 36.9±0.2 | 36.8±0.2* | 36.9±0.2 | 36.7±0.2** |
| H  (/min) | Before | 323±64 | 304±49 | 313±57 | 299±68 | 295±51 | 294±47 | 322±55 | 305±42 | 296±74 |
|  | After | 333±82 | 311±96 | 318±78 | 311±112 | 309±124 | 299±65 | 332±78 | 296±82 | 318±134 |
| P  (/min) | Before | 321±60 | 301±47 | 312±54 | 296±63 | 292±53 | 291±47 | 318±55 | 302±38 | 295±71 |
|  | After | 329±83 | 308±98 | 316±73 | 308±116 | 306±121 | 295±64 | 327±74 | 296±78 | 314±129 |
| R  (/min) | Before | 86±10 | 82±8.5 | 90±10 | 88±14 | 86±11.5 | 85±8 | 89±9.5 | 81±11 | 83±10.5 |
|  | After | 84±17 | 78±16.5 | 86±14 | 88±18.5 | 84±19.5 | 79±10 | 89±12.5 | 78±13.5 | 79±22.5 |
| SpO_2_  (%) | Before | 97.1±2.4 | 97.2±2.1 | 97.4±1.5 | 97.1±2.1 | 97.4±2.5 | 97.6±1.8 | 97.9±1.7 | 97.1±1.6 | 97.3±1.9 |
|  | After | 93.8±2.9* | 94.4±2.5* | 95.2±2.3 | 93.6±3.4* | 92.9±3.9* | 96.1±1.6 | 95.5±2.6 | 95.6±2.2 | 91.9±4.4* |

***Note*:** The indexes of vital signs of MCAO/R model rats before and instantly after perfusion in each subgroup were statistically analyzed by the Student's *t*-test, n=7, Mean ± SD. **p*<0.05，***p*<0.01. T, temperature; H, heart rate; P, pulse; R, respiratory rate; SpO_2_, blood oxygen saturation. H_1_: 1/3 R_ICA_, 30 min, 4 ℃; H_2_: 1/2 R_ICA_, 20 min, 4 ℃; H_3:_ 2/3 R_ICA_, 10 min, 4℃; H_4:_ 2/3 R_ICA_, 20 min, 10℃; H_5:_ 1/2 R_ICA_, 30 min, 10 ℃; H_6:_ 1/3 R_ICA_, 10 min, 10 ℃; H_7:_ 1/3 R_ICA_, 20 min, 15 ℃; H_8:_ 1/2 R_ICA_, 10 min, 15 ℃; H_9:_ 2/3 R_ICA_, 30 min, 15℃. R_ICA_: blood flow rate of the internal carotid artery (ICA) per [minute](D:/Program%20Files/Youdao/Dict/Dict/8.9.6.0/resultui/html/index.html#/javascript:;). "Before" indicates before perfusion; "After" indicates instantly after perfusion.

| **Supplementary Table 3. The effect of IAH on indexes of blood routine tests of MCAO/R model rats in each subgroup.** | | | | | | | | | | |
| --- | --- | --- | --- | --- | --- | --- | --- | --- | --- | --- |
| **Indexes** | **Detection time** | **H_1_** | **H_2_** | **H_3_** | **H_4_** | **H_5_** | **H_6_** | **H_7_** | **H_8_** | **H_9_** |
| RBC (x10^12^/L) | Before | 7.43±0.62 | 7.27±0.49 | 7.41±0.57 | 7.19±0.48 | 7.24±0.51 | 7.32±0.47 | 7.51±0.55 | 7.45±0.42 | 7.36±0.54 |
|  | After | 6.65±0.82 | 6.58±0.99 | 6.98±0.77 | 6.17±1.12* | 6.08±1.24* | 7.14±0.65 | 6.86±0.78 | 7.04±0.87 | 5.82±1.34* |
| HGB  (g/L) | Before | 143.6±10.3 | 152.1±8.5 | 157.4±10.4 | 147.9±11.4 | 154.7±9.5 | 145.8±8.4 | 156.2±9.5 | 146.8±10.7 | 151.6±8.9 |
|  | After | 129.3±17.4 | 139.2±16.8 | 143.1±14.4 | 130.4±18.2 | 126±19.8** | 139.8±10.3 | 144.7±12.6 | 138.1±13.4 | 117.4±22.8** |
| HCT  (%) | Before | 49.16±2.95 | 50.21±2.18 | 49.43±1.52 | 50.14±2.11 | 49.46±2.53 | 48.62±1.85 | 49.94±2.86 | 48.13±1.67 | 49.35±2.91 |
|  | After | 42.51±3.97** | 43.86±3.73** | 44.94±2.55** | 39.82±4.35** | 38.97±4.67** | 45.93±1.63* | 44.24±3.24** | 43.77±2.61** | 37.36±5.12** |
| WBC  (x10^9^/L) | Before | 12.62±2.26 | 12.74±2.15 | 12.54±2.27 | 12.36±2.13 | 12.48±2.19 | 12.12±2.08 | 12.17±1.98 | 12.23±2.34 | 12.85±2.18 |
|  | After | 9.28±3.02* | 10.17±2.84 | 10.52±2.58 | 8.98±3.36* | 8.45±3.62* | 11.61±2.16 | 10.98±2.67 | 10.24±2.39 | 7.78±3.96* |
| Plt  (x10^9^/L) | Before | 1168±83 | 1153±96 | 1146±75 | 1159±84 | 1172±92 | 1184±77 | 1157±68 | 1159±84 | 1159±76 |
|  | After | 951±129** | 974±117** | 989±103** | 935±138** | 926±146** | 1015±96** | 963±119** | 978±105** | 874±167** |

***Note*:** The indexes of blood routine tests of MCAO/R model rats were statistically analyzed using the Student's t-test before and instantly after perfusion in each subgroup. n=7, Mean ± SD. **p*<0.05，***p*<0.01. RBC, the number of red blood cells; HGB, hemoglobin concentration; HCT, hematocrit; WBC, the number of white blood cells; Plt, the number of blood platelet. H_1_: 4°C,1/3 R_ICA_, 30 min; H_2_: 4°C, 1/2 R_ICA_, 20 min; H_3_: 4°C, 2/3 R_ICA_, 10 min; H_4_: 10°C, 2/3 R_ICA_, 20 min; H_5_: 10°C,1/2 R_ICA_, 30 min; H_6_: 10°C, 1/3 R_ICA_, 10 min; H_7_: 15°C, 1/3 R_ICA_, 20 min; H_8_: 15°C,1/2 R_ICA_,10 min; H_9_: 15°C, 2/3 R_ICA_, 30 min; R_ICA_: blood flow rate of the internal carotid artery (ICA) per [minute](D:/Program%20Files/Youdao/Dict/Dict/8.9.6.0/resultui/html/index.html#/javascript:;), estimated as 0.75ml/min. "Before" indicates before perfusion; "After" indicates instantly after perfusion.

| **Supplementary Table 4. The effect of IAH on biochemical indexes of MCAO/R model rats in each subgroup.** | | | | | | | | | | |
| --- | --- | --- | --- | --- | --- | --- | --- | --- | --- | --- |
| **Indexes** | **Detection time** | **H_1_** | **H_2_** | **H_3_** | **H_4_** | **H_5_** | **H_6_** | **H_7_** | **H_8_** | **H_9_** |
| ALB  (g/L) | Before | 36±3 | 34±5 | 33±2 | 32±4 | 35±3 | 34±4 | 31±3 | 32±2 | 35±4 |
|  | After | 33±4 | 32±3 | 31±3 | 29±4 | 31±4 | 32±3 | 29±2 | 30±4 | 28±3** |
| Chol  (mmol/) | Before | 2.31±0.12 | 2.35±0.15 | 2.33±0.13 | 2.34±0.16 | 2.21±0.11 | 2.35±0.15 | 2.28±0.12 | 2.24±0.16 | 2.25±0.13 |
|  | After | 2.49±0.21 | 2.53±0.19 | 2.42±0.15 | 2.54±0.22 | 2.46±0.25* | 2.43±0.11 | 2.39±0.16 | 2.33±0.14 | 2.54±0.28* |
| Ca  (mmol/L) | Before | 2.39±0.14 | 2.32±0.13 | 2.37±0.15 | 2.34±0.11 | 2.36±0.13 | 2.29±0.16 | 2.31±0.15 | 2.35±0.17 | 2.44±0.11 |
|  | After | 2.22±0.16 | 2.15±0.15* | 2.25±0.13 | 2.13±0.17* | 2.12±0.19* | 2.20±0.10 | 2.15±0.16 | 2.21±0.15 | 2.08±0.21** |
| GLU  (mmol/) | Before | 5.73±0.62 | 5.54±0.49 | 5.63±0.57 | 5.49±0.68 | 5.45±0.51 | 5.34±0.47 | 5.72±0.55 | 5.55±0.42 | 5.66±0.54 |
|  | After | 6.73±0.82* | 6.87±0.99** | 6.48±0.77* | 7.17±1.12** | 7.52±1.24** | 5.98±0.65 | 6.57±0.78* | 6.36±0.82* | 7.94±1.34** |
| ALK  (U/L) | Before | 45±7 | 38±10 | 42±8 | 34±9 | 36±12 | 38±6 | 47±9 | 46±6 | 39±7 |
|  | After | 58±13* | 49±11 | 53±10* | 56±16** | 61±17** | 45±8 | 58±11 | 55±9* | 66±19** |
| ALT  (U/L) | Before | 35±9 | 36±8 | 39±5 | 32±10 | 28±10 | 34±5 | 41±7 | 42±6 | 29±12 |
|  | After | 53±12** | 52±11** | 50±10* | 53±14** | 52±17** | 43±6* | 53±11* | 52±9* | 56±20** |
| AST  (U/L) | Before | 32±7 | 31±10 | 34±6 | 32±11 | 31±12 | 31±8 | 39±9 | 40±7 | 30±10 |
|  | After | 48±11** | 50±12** | 45±9* | 52±13** | 53±15* | 41±7* | 52±10* | 51±8* | 55±18** |
| CK  (U/L) | Before | 46±10 | 42±8 | 50±10 | 48±14 | 46±11 | 45±8 | 49±9 | 41±11 | 53±10 |
|  | After | 112±27** | 108±26** | 98±24** | 122±28** | 134±29** | 89±20** | 105±22** | 95±23** | 149±32** |
| BUN  (mmol/) | Before | 6.54±1.21 | 6.76±1.18 | 6.94±1.05 | 6.91±1.24 | 6.78±1.21 | 6.79±1.17 | 6.81±1.12 | 6.62±1.13 | 6.57±1.27 |
|  | After | 7.16±1.62 | 7.25±1.53 | 7.32±1.46 | 7.67±1.75 | 7.55±1.72 | 7.09±1.42 | 7.35±1.58 | 7.04±1.49 | 7.43±1.86 |
| CREA  (μmol/L) | Before | 55±9 | 56±8 | 49±5 | 46±11 | 48±14 | 44±6 | 47±9 | 46±13 | 51±7 |
|  | After | 57±13 | 54±11 | 53±10 | 58±14 | 60±15 | 49±16 | 53±18 | 52±15 | 62±16 |

***Note*:** The biochemical indexes before and after perfusion in each subgroup were analyzed by the Student's t-test (n=7, Mean ± SD). **p*<0.05，***p*<0.01. ALB, albumin; Chol, cholesterol; Ca, serum calcium; GLU, blood glucose; ALK, alkaline phosphates; ALT, alanine aminotransferase; AST, aspartate aminotransferase; CK, creatine kinase; BUN, blood urea nitrogen; CREA, creatinine. H_1_: 4°C,1/3 R_ICA_, 30 min; H_2_: 4°C, 1/2 R_ICA_, 20 min; H_3_: 4°C, 2/3 R_ICA_, 10 min; H_4_: 10°C, 2/3 R_ICA_, 20 min; H_5_: 10°C,1/2 R_ICA_, 30 min; H_6_: 10°C, 1/3 R_ICA_, 10 min; H_7_: 15°C, 1/3 R_ICA_, 20 min; H_8_: 15°C,1/2 R_ICA_,10 min; H_9_: 15°C, 2/3 R_ICA_, 30 min; R_ICA_, blood flow rate of the internal carotid artery (ICA) per minute. "Before" indicates before perfusion; "After" indicates after perfusion.
